# Controllable band structure and topological phase transition in two-dimensional hydrogenated arsenene

**DOI:** 10.1038/srep20342

**Published:** 2016-02-03

**Authors:** Ya-ping Wang, Wei-xiao Ji, Chang-wen Zhang, Ping Li, Feng Li, Miao-juan Ren, Xin-Lian Chen, Min Yuan, Pei-ji Wang

**Affiliations:** 1 School of Physics and Technology, University of Jinan, Jinan, Shandong, 250022, People’s Republic of China

## Abstract

Discovery of two-dimensional (2D) topological insulator such as group-V films initiates challenges in exploring exotic quantum states in low dimensions. Here, we perform first-principles calculations to study the geometric and electronic properties in 2D arsenene monolayer with hydrogenation (HAsH). We predict a new σ-type Dirac cone related to the p_x,y_ orbitals of As atoms in HAsH, dependent on in-plane tensile strain. Noticeably, the spin-orbit coupling (SOC) opens a quantum spin Hall (QSH) gap of 193 meV at the Dirac cone. A single pair of topologically protected helical edge states is established for the edges, and its QSH phase is confirmed with topological invariant Z_2_ = 1. We also propose a 2D quantum well (QW) encapsulating HAsH with the *h*-BN sheet on each side, which harbors a nontrivial QSH state with the Dirac cone lying within the band gap of cladding BN substrate. These findings provide a promising innovative platform for QSH device design and fabrication operating at room temperature.

Two-dimensional (2D) topological insulators (TIs), known as quantum spin Hall (QSH) insulators, have attracted significant researches interest in condensed matter physics and materials science. The unique characteristic of 2D TI is generating a gapless edge state inside the band insulating gap, in which the edge state is topologically protected by time-reversal symmetry (TRS)^1^,^2^ and more robust against backscattering than the 3D TI, making 2D TIs better suited for coherent spin transport related applications. The prototypical concept of QSH insulator is first proposed by Kane and Mele in graphene^3^,^4^, in which the spin-orbit coupling (SOC) opens a band gap at the Dirac point. However, the associated gap due to rather weak second-order effective SOC is too small (~10^–3 ^meV), which makes the QSH state in graphene only appear at an unrealistically low temperature. Quantized conductance through QSH edge states have been experimentally demonstrated in HgTe/CdTe^5^,^6^ and InAs/GaSb^7^,^8^ quantum-wells (QWs), showing an interesting in further experimental studies and possible applications.

Currently, there is a great interest in searching for new QSH insulators in 2D materials with controllable quantum phase transitions and tunable electronic and spin properties. Remarkably, the orbital filtering effects (OFE) in engineering the band feature has been received intense attentions in designing QSH insulators. For instance, stanene^9^ has a small band gap of 0.1 eV where the p_z_ orbital dominates the effective low-energy band structure. Through hydrogenation, the SOC can be confined both on the p_x_ and p_y_ orbitals of stanene, enhancing its band gap to 0.3 eV. In fact, the band gap enhancement in QSH phase can also be realized by decorating organic molecule ethynyl on stanene film^10^. The strong SOC can be sufficed by bismuth element, which drives QSH and QAH states^11^. More recently, an approach to design a large-gap QSH state on a semiconductor surface by a substrate orbital selection process is also proposed^12^. These demonstrate that OFE is an effective way to enhance QSH effect in 2D materials with s and p orbitals dominating the conduction and valence bands.

Group-V honeycomb structures have recently attracted interests as novel 2D materials with intriguing electronic properties. Interestingly, the monolayer form of black phosphorous, phosphorene (α-P), has been reported experimentally to have a direct band gap and high carrier mobility, which can be exploited in the electronics^13^,^14^. The Bi or Sb ultrathin films^15^,^16^,^17^,^18^, another 2D group-V films with the strongest SOC, have been proposed to harbor large-gap QSH phases, ample to applications at room temperature. More recently, arsenene in α and β phases has been proposed to be energetically stable^19^,^20^,^21^,^22^. These materials with high mechanical stretchability, which can reversibly withstand extreme mechanical deformation, are useful to stretchable display devices, broadband photonic tuning and aberration-free optical imaging^19^,^20^,^21^. More importantly, we find the pristine arsenene can be a QSH insulator, but its band gap is relative small, unfavorable to possible room temperature applications^22^. However, the band topology of hydrogenated arsenene (HAsH) have not been reported up to date. It is thus reasonable to ask whether or not HAsH becomes a nontrivial QSH insulator, which maybe largely widens its application in spintronics.

In this work, based on first-principles calculations, we predict a σ-type Dirac cone at the K point in HAsH. The key is the OFE from decorated hydrogen atoms, in which the out-of-plane p_z_ is filtered from p orbitals, forming sp^2^ hybridization, in analogy to planar graphene. A QSH phase with a band-gap as large as 193 meV at the Fermi level is obtained, available to practical application at room temperature. A single pair of topologically protected helical edges is established, and its QSH phase is confirmed with Z_2_ = 1. We also propose a QW encapsulating HAsH between the BN sheet on each side, maintaining a nontrivial QSH state with the Dirac cone lying within the band gap of cladding BN substrate. These results provide an ideal platform for development of high-performance electronic devices in spintronics.

## Methods

First-principles calculations based on density-functional theory (DFT)^23^ are performed by the Vienna ab initio simulation package^24^, using the projector- augmented-wave potential. The exchange-correlation functional is treated using the Perdew-Burke-Ernzerhof (PBE)^25^ generalized-gradient approximation. The energy cutoff of the plane waves is set to 500 eV with the energy precision of 10^–5 ^eV. The Brillouin zone (BZ) is sampled by using a 9 × 9 × 1 Gamma-centered Monkhorst–Pack grid, and the vacuum space is set to 30 Å to minimize artificial interactions between neighboring slabs. All structures are fully optimized, including cell parameters and atomic coordinates, until the residual forces are less than 0.01 eV/Å. The SOC is included in the self-consistent calculations of electronic structure.

## Results and Discussion

**Figure 1:**
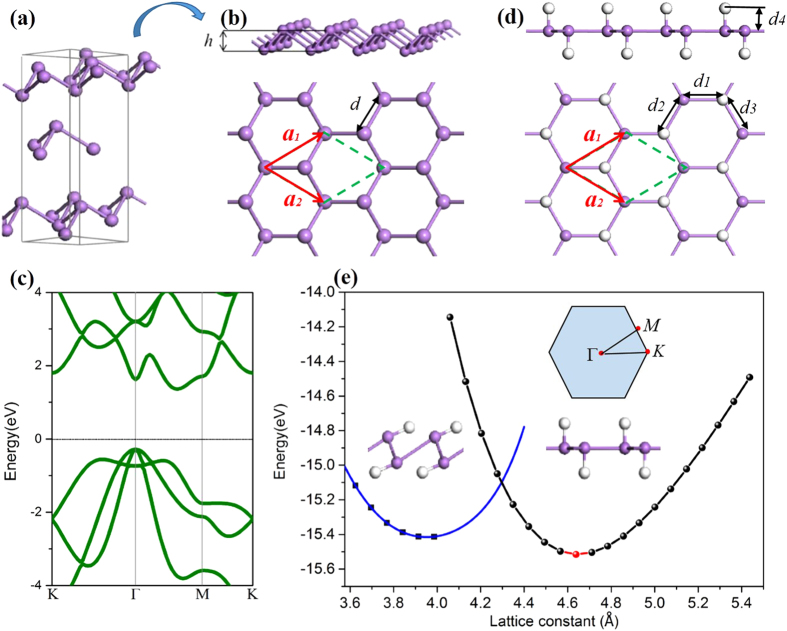
(**a**) Side view of the rhombohedrally (ABC) stacked layered structure of gray arsenic; (**b**) The side and top views of buckled honeycomb structure from gray arsenic; (**c**)The band structure of arsenene; (**d**) d_1_–d_4_ are the various bond lengths of corresponding position, the buckling heights was also marked as h; (**e**) Total energy with respect to lattice constant. The insert is Brillouin zone. The violet and silver balls denote arsenic and hydrogen atoms, respectively.

Bulk As has four allotropes, and the most stable one is gray As^20^, which is rhombohedral with two atoms per primitive cell. Thus, it can be viewed as a stacking of the bilayers along the [111] direction, as shown in Fig. 1(a). Unlike the planar graphene, the arsenene has a buckled configuration with a buckling distance h = 1.39 Å and bond length d = 2.51 Å (Fig. 1(b)), in consistent with that of Ref. 19 and 20. Figure 1(c) displays the calculated band structures of arsenene, which is indirect-gap semiconductor with a gap of 1.64 eV at the Fermi level. In this respect, its valence band maximum (VBM) locates the Γ point, while conduction band minimum (CBM) on M-Γ path, in agreement with the previous results^20^.

Hydrogenation has been proven to be an efficient way in engineering the electronic properties in 2D materials^10^,^26^,^27^,^28^,^29^,^30^,^31^,^32^. Thus, we saturate the uncoordinated As atoms with hydrogen atoms alternating on both sides of As sheet, in which *d*_1_−*d*_4_ represents the various bond lengths between As atoms (Fig. 1(d)). In sharp contrast to the graphane and silicane^33^,^34^, the structural relaxation make the σ-bond direction of As-H atoms tiled and finally perpendicular to As-As bonds, indicating hydrogen-induced arsenic dimerization with respect to tensile strain, as listed in Table 1. Figure 1(e) gives the total energy per unit cell as a function of lattice constant *a*. Interestingly, it displays two local minima in energy, where refer to the corresponding stable phases as buckled and planar states in order to highlight the size of buckling or interlayer distance in these two distinct states (Table 1).

**Figure 2:**
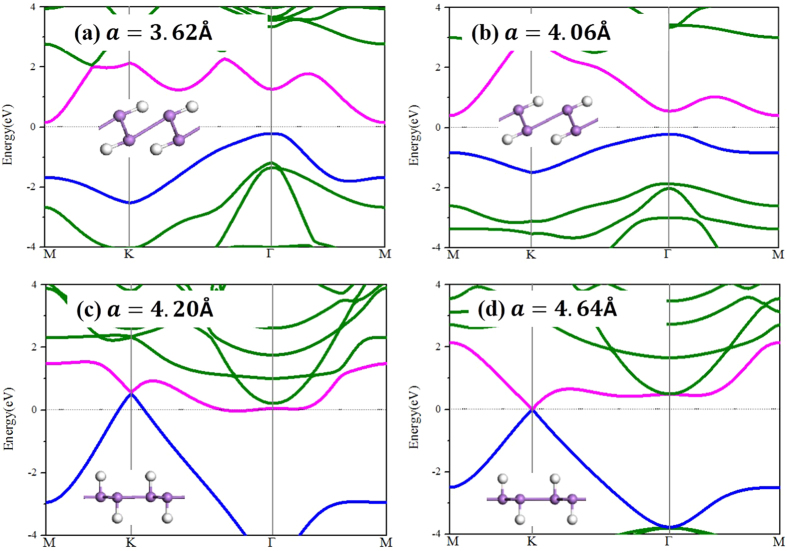
(**a–d**) The band structures with respect to different lattice constant, the insets are side views of the configurations. The violet and silver balls denote arsenic and hydrogen atoms, respectively. Magenta and blue bands represent the conduction and valence bands near the Fermi surface.

External strains can drastically change the geometric structures, and thus influence the electronic properties of HAsH correspondingly. As shown in the insert of Fig. 2, when applying the tensile strain, the As–As dimerization occurs clearly, leading to an indirect-direct gap transition. Further increasing to 4.20 Å, the As-As dimerization is suppressed, along with the arsenene plane becoming flat. In this case, a clear band crossing appears at the K point (Fig. 2(c)), as compared with the original indirect-gap semiconductor feature. If the lattice constant reaches 4.64 Å, it becomes completely flat with As–As bonds being equivalent, due to the complete dimerization breaking (Fig. 2(d)). The two energy bands crosses linearly at the K (and K′ = −K) point, suggesting the existence of Dirac-cone feature without SOC. Thus it can be considered as a gapless semiconductor, or alternatively, as a semi-metal with zero density of states at the Fermi level. Further increasing the lattice constant, the Dirac-cone preserves with the crossing at the Γ point shifting toward higher energy, which can be attributed to the simultaneous release of out-of-plane buckling in arsenene. For instance, if the lattice parameter increases as much as 28%, i.e., from a = 3.62 Å to a = 4.64 Å, the As–As bonds simply elongate from 2.48 Å to 2.68 Å, only corresponding to a stretch of 8%. As expected, it is geometric transition from buckling to planar one that plays a key role in the presence of the Dirac cone.

**Figure 3:**
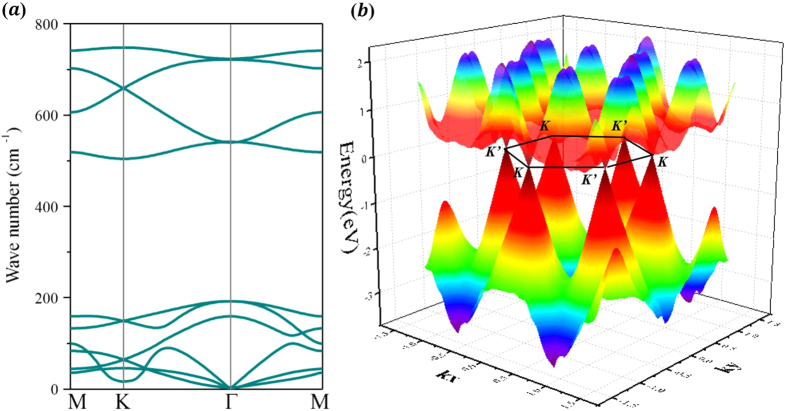
(**a**) Phonon dispersion of hydrogenated arsenene. (**b**) The 3D band structure of hydrogenated arsenene, around the Dirac point at a = 4.64 Å.

To prove the dynamic stability of this structure (a = 4.64 Å), we present the calculated phonon spectrum in Fig. 3(a). All phonon branches are positive, which indicates that this structure is kinetically stable. Moreover, Fig. 3(b) shows 3D band structure around the Dirac point at a = 4.64 Å. we can see two Dirac cones located at the K and K′ = −K points, similar valley symmetry as in graphene^3^,^4^. However, the energy spectrum here is no longer electron-hole symmetric, thus neither the scattering mechanism nor transport properties will be identical for the electron and hole doping cases. The linear dispersion holds up to 2.0 eV for holes, while the massless electrons acquire mass rapidly away from the K point, demonstrating that it is more promising for making unipolar field effect devices than for making ambipolar ones with graphene.

**Figure 4:**
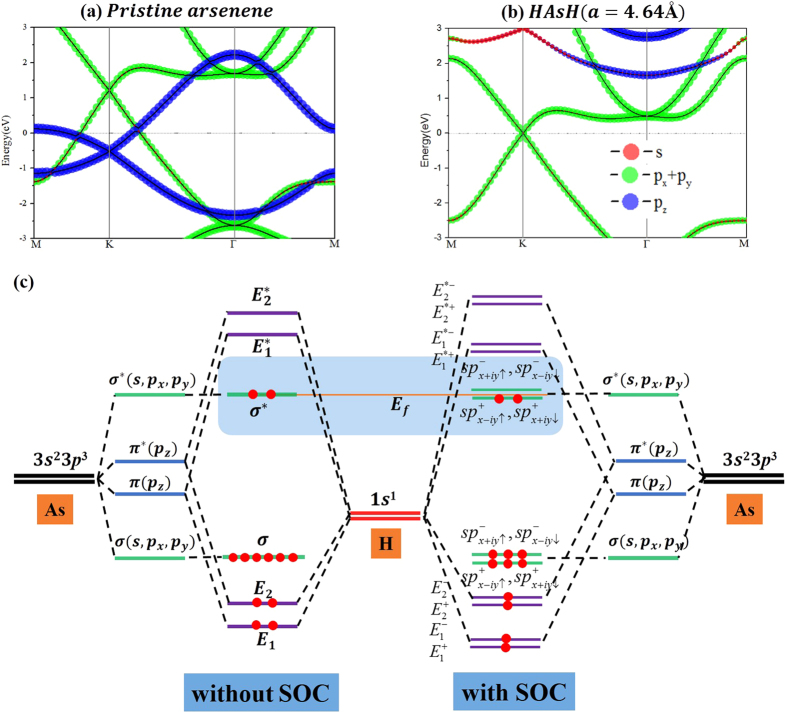
(**a**) Band structure of monolayer arsenene with a = 4.64 Å. (**b**)the aforementioned system but with H saturated, and the contributions from s, p_x,y_ and p_z_ orbitals, while red, green and blue dots represent the contribution of s, p_x,y_, and p_z_ orbitals. (**c**) Schematic diagram of the electronic transition. The line thickness represents orbital degeneracy, whereas the red dot represents an occupied electron. The Fermi levels are at energy zero.

Now, we highlight the importance of the OFE^9^,^10^,^11^,^12^ in determining the origin of Dirac cone at a = 4.64 Å. In the absence of hydrogen atoms (Fig. 4(a)), one can see two Dirac cones located at the K point. By projecting the component of bands, the upper Dirac cone (green circles) originates from the p_x,y_ orbitals, whereas the lower one (blue circles) originates from p_z_ orbital. Through fully-hydrogenation, the As-p_z_ orbital bond to H-s orbital is filtered, making the low Dirac cone disappear (Fig. 4(b)). Thus, the remaining Dirac cone mainly comes from As-p_x,y_ orbitals rather than from p_z_ orbital, as illustrated by the size of the circles near the Fermi energy. In this case, the p_x,y_ orbitals form σ bonds between As-As atoms, demonstrating an in-plane Dirac cone feature. Figure 4(c) further illustrates the schematic plot of energy level dispersion. As expected, the chemical bonding of As-As atoms makes the s and p_x,y_ orbitals forming the bonding and anti-bonding states, in the energy consequence of σ < π < π* < σ*, The planar honeycomb geometry separates in-plane (p_x,y_) and out-plane (p_z_) orbitals, forming their own Dirac cones with σ and π characters, respectively (Fig. 4(a)). The hybridization between the π orbitals and induced H-1s orbital suppresses the π-type Dirac cone, leaving the σ-type Dirac cone intact. In addition, the Fermi level is raised by introduced hydrogen atoms, and thus forming the semi-metallic states.

**Figure 5:**
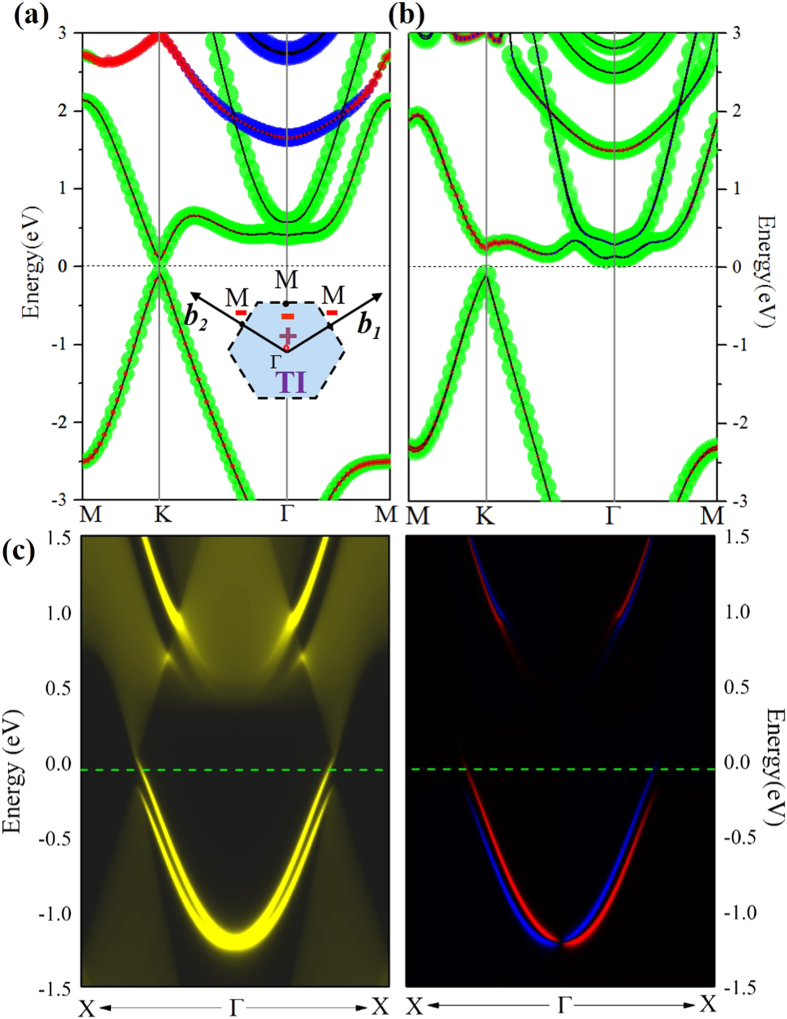
(**a,b**) Band structures of hydrogenated arsenene with SOC using PBE and HSE06. Here, the insert is Brillouin zone. (**c**) Electronic structure of helical edge states of hydrogenated arsenene, the left subpanel shows the total density of states while the right subpanel shows the corresponding spin polarization in two channels.

The As atom has an intrinsically larger SOC than a carbon atom, which may lead to many intriguing quantum properties in arsenene, such as the QSH effect^3^,^4^,^35^, in addition to its massless Dirac fermion as in graphene. To confirm this, we focus on the effect of SOC on band structures at 4.64 Å, as displayed in Fig. 5(a). One can see that the degeneracy at the Dirac points is lifted. The valence bands are downshifted whereas the conduction bands are upshifted, forming a large band gap of 193 meV by SOC, as illustrated in Fig. 4(c). As observed in previously reported 2D TIs like phosphorene^36^, ZrTe_5_, HfTe_5_^37^, and GaSe^38^, the SOC-induced band-gap opening at the Fermi level is a strong indication of the existence of topologically nontrivial phases. We have carried out test calculations based on the hybrid functional HSE06 to assess the robustness of our results. As shown in Fig. 5(b), the band gap around Dirac point is increased to 339 meV, but the band character is not changed, which is agreement with our PBE results.

To identify the nontrivial band topology in 2D HAsH, we calculate the topologically invariant Z_2_ number (*γ*) following the approach proposed by Fu and Kane^39^, due to the presence of inversion symmetry. Here, the invariants *v* can be derived from the parities of wave function at the four time-reversal-invariant momenta (TRIM) points K_i_, namely one Γ point and three M points in the Brillouin zone, as illustrated in the insert of Fig. 5(a). The topological index ν are established by


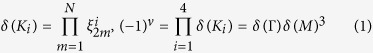


where *δ* is the product of parity eigenvalues at the TRIM points, *ξ* = ±1 are the parity eigenvalues and *N* is the number of the occupied bands. According to the Z_2_ classification, *ν* = 1 characterizes a QSH insulator, whereas *ν* = 0 represents a trivial band topology. As expected, in the equilibrium state, the products of the parity eigenvalues at these three symmetry points: M(0.0, 0.5), M(0.5, 0.5) and M(0.5, 0.0) are both −1, while at Γ(0.0, 0.0) and displays +1, yielding a nontrivial topological invariant Z_2_ = 1.

The SOC induced band gap opening near the Fermi level indicates possible existence of 2D TI state that are helical with the spin-momentum locked by TRS. To check this, we calculated the topological edge states of HAsH by the Wannier90 package^40^. We construct the maximally localized Wannier functions (MLWFs) and fit a tight-binding Hamiltonian with these functions. Then, the edge Green’s function^41^ of a semi-infinite HAsH is constructed and the local density of state (LDOS) is calculated, as shown in Fig. 5(b). Clearly, all the edge bands are seen to link the conduction and valence bands and span the 2D band energy gap, yielding a 1D gapless edge states. Besides, the counter-propagating edge states exhibit opposite spin polarizations, in accordance with the spin-momentum locking of 1D helical electrons. All the above results consistently indicate that hydrogenated arsenene is an ideal 2D TI.

**Figure 6:**
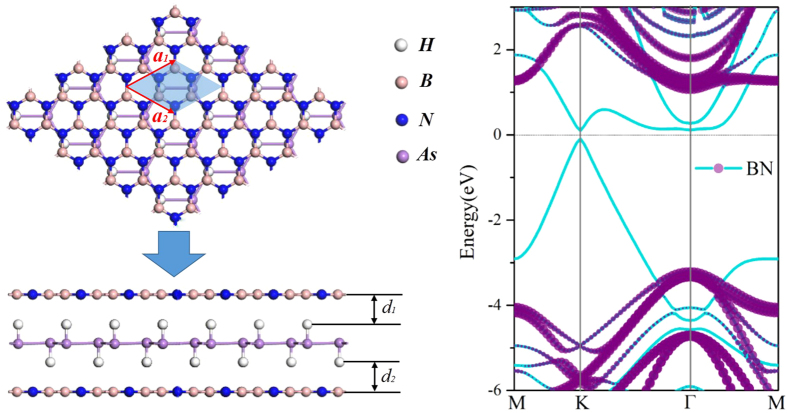
Crystal structures of QW consisting of HAsH monolayer sandwiched *h*-BN sheet on each side from the top and side view for (**a**), as well as the corresponding band structure with SOC for (**b**). The dotted lines with the pink color originate from the *h*-BN sheet.

The substrate materials are inevitable in device application, thus the free-standing HAsH monolayer should eventually be deposited or grown on a substrate. As a 2D large-gap insulator with a high dielectric constant, the *h*-BN sheet has been successfully used as the substrate to grow graphene or assemble 2D stacked nanodevices^42^,^43^. Considering that the HAsH surface is chemically active, we adopt the encapsulation technology, which has been used in Flip-Chip^44^, to encapsulate HAsH monolayer forming a hybrid QW from the degradation effect by the environmental gases, which, if not prevented, would destroy the QSH states. Figure 6(a) show the QW structure of HAsH monolayer sandwiched on 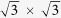 BN sheet, where the lattice mismatch is only about 6.3%. After full relaxation with the van der Waals (vdW) forces^45^, HAsH almost retain the original structure with a distance between the adjacent *h*-BN layers of 2.38 Å. The calculated binding energy is about −0.77 eV per unit cell, showing that they are typical van der Waals interactions. The calculated band structure with SOC is shown in Fig. 6(b). In these weakly coupled QW structure, the HAsH monolayer remains semiconducting, there is essentially no charge transfer between the adjacent layers, and the states around the Fermi level are dominantly contributed by HAsH. If we compare the bands of HAsH with and without the cladding BN sheet, little difference is observed. Evidently, the robustness of QSH effect can be preserved in this QW structure.

## Conclusions

In summary, based on first-principles calculations, we predict a new σ-type Dirac cone related to the p_x,y_ orbitals of As atoms in HAsH, dependent on in-plane tensile strain. The key is to separate the in-plane p_x,y_ and out-of-plane p_z_ orbitals via hydrogenation and strain. Noticeably, spin-orbit coupling (SOC) can open a nontrivial QSH gap of 193 meV at the Dirac cone. A single pair of topologically protected helical edge states is established for the edge of HAsH, and its QSH states are confirmed with topological invariant Z_2_ = 1. We propose a QW encapsulating HAsH between the *h*-BN sheet on each side, maintaining a nontrivial QSH state with the Dirac cone lying within the band gap of cladding BN sheet. These findings provide a promising innovative platform for QSH device design and fabrication operating at room temperature.

## Additional Information

**How to cite this article**: Wang, Y.-P. *et al*. Controllable band structure and topological phase transition in two-dimensional hydrogenated arsenene. *Sci. Rep*. **6**, 20342; doi: 10.1038/srep20342 (2016).

## Figures and Tables

**Table 1:** The geometric structures including buckling height between As atoms (h), As–As bond lengths, As-H bond lengths, as well as the whole band gap of corresponding band structure.

Structure	As-As			As-H	*h*(Å)	Gap(eV)
	*d*_1_	*d*_2_	*d*_3_	*d*_4_		
buckled-As	2.51	2.51	2.51	–	1.39	1.64
HAsAsH-3.62	3.00	2.48	2.48	1.55	1.59	0.36
HAsAsH-4.06	3.26	2.59	2.59	1.55	1.49	0.62
HAsAsH-4.20	2.44	2.44	2.41	1.54	0.04	0.00
HAsAsH-4.64	2.68	2.68	2.68	1.54	0.00	0.00

Here, the sign ‘-’ means the absence of the value of the corresponding position.
